# Surgical outcomes and complication rates in severe scoliosis: a systematic review

**DOI:** 10.1051/sicotj/2025050

**Published:** 2025-09-19

**Authors:** Luthfi Gatam, Phedy Phedy, Harmantya Mahadhipta, Syafrudin Husin, Asrafi Rizki Gatam, Pranajaya Dharma Kadar, Karina Sylvana Gani, Mitchel Mitchel, Erica Kholinne

**Affiliations:** 1 Gatam Institute, Eka Hospital Indonesia; 2 Orthopedic and Traumatology Department, Fatmawati Hospital Jakarta Indonesia; 3 Orthopedic and Traumatology Department, Premier Bintaro Hospital Indonesia; 4 Department of Orthopaedics and Traumatology, Faculty of Medicine, Universitas Sumatera Utara Medan Indonesia; 5 Faculty of Medicine, Universitas Trisakti Jakarta Indonesia

**Keywords:** Severe scoliosis, Non-osteotomy, Ponte Osteotomy, Smith-Peterson osteotomy, Vertebral column resection

## Abstract

*Background*: Correcting severe scoliosis is challenging due to curve rigidity and risks to cardiopulmonary and neurologic function. Osteotomy techniques offer greater correction but carry higher complication rates, while non-osteotomy methods may be safer but less effective. This systematic review compares outcomes between osteotomy and non-osteotomy approaches in treating severe idiopathic scoliosis. *Methods*: A systematic search was conducted in PubMed, EMBASE, and the Cochrane Library using MeSH terms related to “idiopathic adolescent scoliosis”, “AIS”, “severe scoliosis”, and “surgical outcome”. The review followed PRISMA guidelines. *Results*: An initial search yielded 565 studies, of which 23 studies (*n* = 932 patients) met the inclusion criteria. The Vertebral Column Resection (VCR) group achieved the greatest spinal correction, with a mean Cobb angle of 106.7 ± 9.7° and a correction rate of 62.1%, but also had the highest complication rate at 24%. Non-osteotomy methods provided similar correction (107.0 ± 9.1°, 61.5%) with a slightly lower complication rate of 19.6%. The Ponte osteotomy group had the lowest complication rate (4%) with a moderate level of correction (107.4 ± 10.5°, 60.3%). In terms of functional outcomes, the non-osteotomy group reported the highest SRS-22r scores, averaging 4.3. *Conclusion*: VCR offers the most significant curve correction, but with a higher complication rate. Ponte osteotomy provides a safer alternative with acceptable clinical outcomes. In contrast, non-osteotomy techniques strike a balance between correction and risk, with favorable functional results.

## Introduction

Scoliosis affects 0.5–5% of the adolescent population, with an idiopathic incidence of 80% [1, 2]. Severe scoliosis is characterized by a major curve exceeding 90° and less than 25% correction on bending films [3]. Delayed diagnosis, treatment, and aggressive scoliosis patterns can result in significant curve progression and are often linked to psychological disorders [4]. Various correction methods have been reported to date, including spinal fusion, posterior instrumentation with anterior release, combined anterior-posterior procedures with halo traction, vertebral column resection, combined anterior and posterior instrumentation, and Ponte osteotomies [5]. Spinal osteotomy is an effective treatment for severe spinal deformities, achieving correction rates between 51% and 69%. However, it carries a higher risk of complications, including significant blood loss, infection, cerebrospinal fluid leakage, and implant failure [6]. The non-osteotomy approach is less invasive and associated with lower perioperative morbidity, as it corrects spinal deformity without bone resection. However, its effectiveness may be limited in achieving optimal correction in rigid curves [7]. While both osteotomy and non-osteotomy surgical strategies are used in clinical practice, current literature lacks direct comparative studies assessing the complication profiles and clinical outcomes of these two approaches in the context of severe scoliosis.

This systematic review aims to evaluate and compare the clinical outcomes of osteotomy versus non-osteotomy for treating severe scoliosis. The secondary objective is to compare the complications and satisfaction rates of these two treatment approaches. The findings will guide clinical decision-making and optimize patient outcomes.

## Materials and methods

This study was conducted in accordance with the Preferred Reporting Items for Systematic Reviews and Meta-Analysis (PRISMA) guidelines. The selection criteria focused on studies comparing non-osteotomy and osteotomy in patients with severe scoliosis, reporting on outcomes such as postoperative Cobb angle, correction rate, intraoperative estimated blood loss (EBL), functional outcomes, complications, and satisfaction rate.

### Bibliographic research protocol

A literature search for eligible studies was conducted on March 22, 2025, using the Cochrane Library, EMBASE, and PubMed databases.

### Eligibility articles and search strategy with information sources

All included studies contained original data, were published in English, and had a follow-up period of at least 12 months. We also examine the reference list of the included studies to ensure optimal research. The search engines were used to locate studies that combined the terms “(“idiopathic adolescent scoliosis”, “AIS”, “severe scoliosis”, and “surgical outcome”)”. The PubMed search was conducted using the following query: (((idiopathic adolescent scoliosis) OR (AIS)) AND (severe scoliosis)) AND (surgical outcome).

### Inclusion and exclusion criteria

This study used the PICO (population, intervention, comparison, and outcomes) model.

Participants: patients with severe scoliosis with preoperative Cobb angle ≥ 90°, regardless of age and sex.

Intervention: osteotomy technique in spine surgery for severe scoliosis, including Ponte osteotomy (posterior column osteotomy), Smith-Peterson osteotomy, and vertebral column resection

Comparison: non-osteotomy technique

Outcomes: postoperative Cobb angle, correction rate, intraoperative estimated blood loss (EBL), functional outcomes, complications, and satisfaction rate.

In this study, we divided the participants into three groups: the non-osteotomy group, the Ponte-Smith osteotomy group, and the vertebral column resection (VCR) group. The exclusion criteria were studies involving non-idiopathic scoliosis, nonsurgical methods, and extremely severe scoliosis with a Cobb angle greater than 130°.

Using standardized forms, duplicates were removed, and then titles and abstracts were screened by two reviewers (M and KSG). They then thoroughly evaluated the full texts of the potential studies to confirm their eligibility.

### Quality assessment and risk of bias assessment

**Figure 1:**
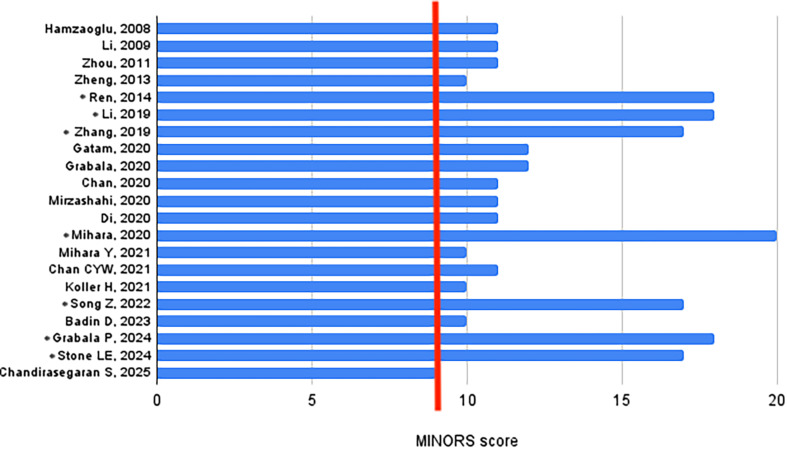
MINORS Quality Assessment of the 14 studies was non-comparative, and seven studies (indicated with asterisks) were comparative. The vertical red line represents the cutoff point for noncomparative studies considered high quality. MINORS, Methodological Index for Non-Randomized Studies.

**Figure 2:**
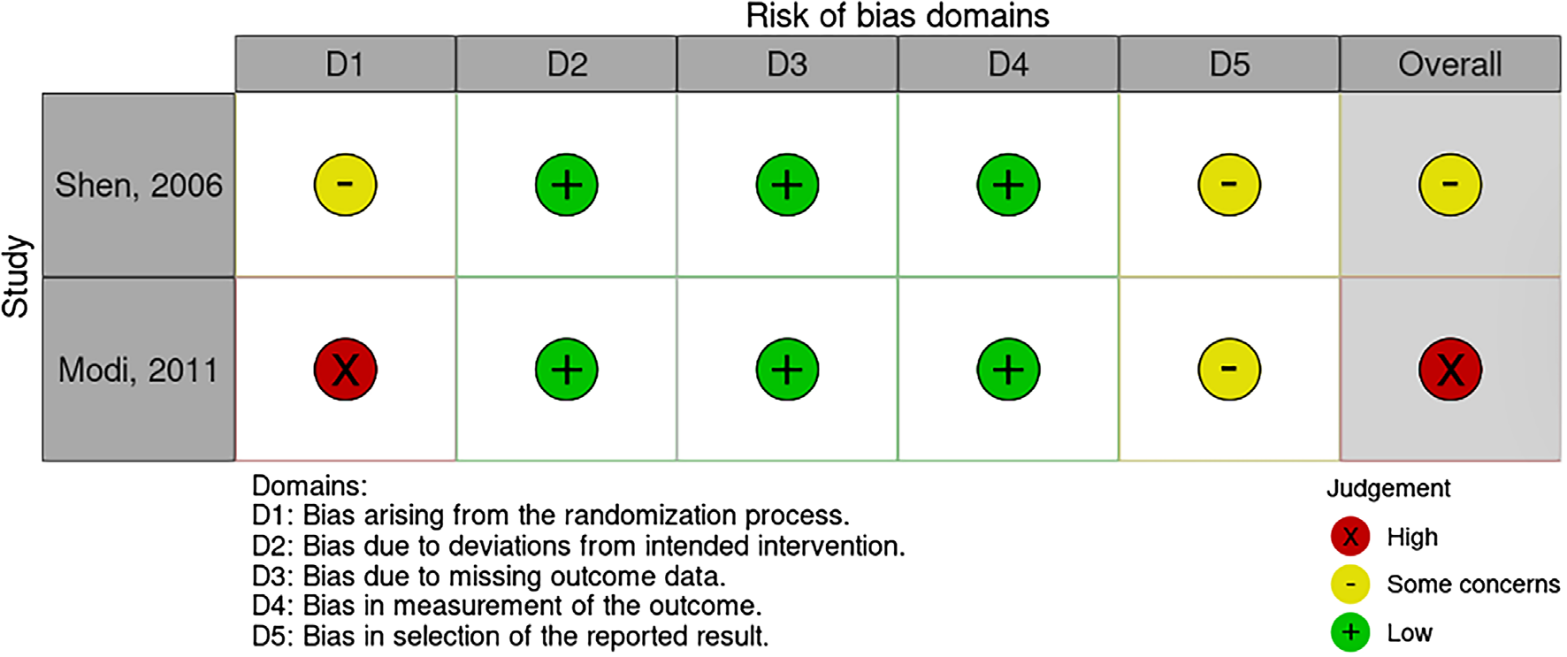
Risk of bias of the included studies.

Two authors (M and KSG) independently reviewed the search results. Studies were initially screened by title and abstract, with full texts of the relevant articles obtained and independently reviewed by both authors. Disagreements between the two authors were resolved through consensus and discussion with a third author (EK). The risk of bias was assessed using the Methodological Index for Non-Randomized Studies (MINORS) score for non-randomized studies and the Cochrane Risk of Bias (RoB) 2.0 assessment tool for randomized studies [7, 8]. The MINORS score allows 16 points for non-comparative studies and 24 points for comparative studies. High-quality studies were defined as those with scores above 60%, 9 out of 16 for non-comparative studies and 14 out of 24 for comparative studies. According to the RoB 2.0 Cochrane tool, which contains five domains (randomization, process, deviations from the intended interventions, missing outcome data, measurement of the outcome, and selection of the reported result), the risk of bias was categorized as high, low, or some concern. The 21 nonrandomized studies comprised 14 noncomparative and seven comparative studies (Figure 1). These studies are considered high-quality studies according to MINORS criteria. Two randomized studies were assessed using the RoB 2 assessment, and one study is of moderate risk of bias, while the other is at high risk of bias (Figure 2).

### Data extraction and analysis

Two authors (M and KSG) independently extracted descriptive data from the selected studies, including the first author, publication year, study design, sample size, follow-up duration, and outcome measures.

**Figure 3:**
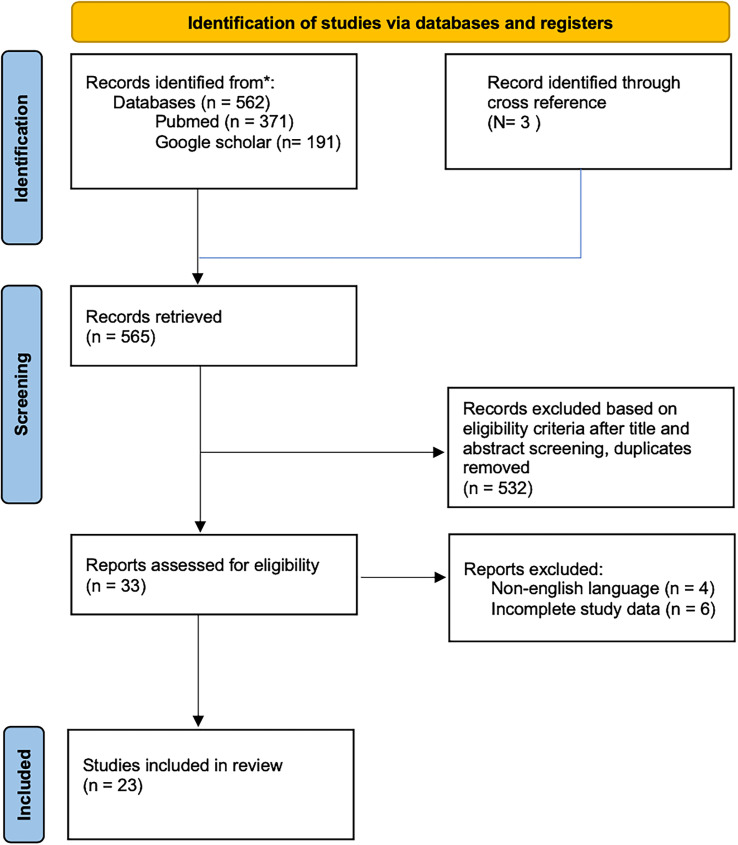
Preferred Reporting Items for Systematic Review and Meta-Analysis (PRISMA) diagram.

Data were extracted from each study’s text, figures, tables, and supplementary files. The extracted data included (1) study and patient characteristics; (2) mean follow-up time; (3) mean preoperative and postoperative Cobb angle; (4) correction rate; (5) intraoperative estimated blood loss; (6) functional outcome score: Scoliosis Research Society-22 Revised (SRS-22R); (7) satisfaction and complication rates. Discrepancies in data extraction were resolved through discussion with a third author. Studies with missing or unextractable data were excluded. A short narrative synthesis was performed, and studies were grouped based on inclusion criteria. The process of study selection is using the PRISMA (Preferred Reporting Items for Systematic reviews and Meta-Analyses) guideline and detailed in Figure 3 [9].

The initial search identified 565 studies, of which 532 were excluded due to duplication or failure to meet the criteria. After screening, 33 were eligible for review. Four studies were excluded because the journals in which they were published were not in the English language, and six studies were excluded due to incomplete study data. Twenty-three studies are included in this systematic review.

## Result

### Characteristics and demographics

A total of 932 cases with severe idiopathic scoliosis were included in this study. The mean age of patients was 16.6 ± 13.1 years. Patients with severe idiopathic scoliosis in the non-osteotomy group, based on 13 studies, were followed up for an average of 38.8 ± 13.3 months. The Smith-Petersen group had a follow-up duration of 70 ± 6.9 months, whereas the VCR group was followed for 46 ± 7.9 months. A total of 639 patients underwent non-osteotomy, 88 patients underwent Smith-Peterson Osteotomy, and 161 patients underwent VCR. The detailed characteristics, demographics, treatment techniques, preoperative, and postoperative are provided in Table 1.

**Table 1:** Characteristics and demographics of the studies.

No	Author, year	Study design	Level of study	Operative technique	Sample size (M/F)	Age (yr)	Follow-up time (months)
*Non-osteotomy*							
1	Shen, 2006 [10]	Retrospective	III	Two-stage combined APSF	4/8 (12)	13.7 ± 3.6	39.6
				One-stage combined spinal fusion	5/7 (12)	15.2 ± 3.6	39.6
2	Hamzaoglu, 2008 [11]	Retrospective	III	Halo-femoral traction and posterior only pedicle screw instrumentation	4/11 (15)	17.8	56
3	Li, 2009 [9]	Retrospective	III	Single-staged APSF	6/25 (31)	15.9	42
4	Zheng, 2013 [12]	Retrospective	III	PSF with a pedicle screw-only construct	11/10 (21)	15	>24
5	Ren, 2014 [13]	Retrospective	III	Anterior release with temporary posterior internal distraction, followed by posterior fusion and instrumentation	8/9 (17)	17.7 ± 2.9	28.1 ± 5.3
6	Mihara, 2020 [14]	Retrospective	III	Single-staged PSF	6/65 (71)	16.1 ± 5.8	N/A
7	Mirzashahi, 2020 [15]	Retrospective	III	Single-staged posterior-only approach	8/15 (23)	16.2	37.0 ± 7.6
8	Chan, 2020 [16]	Retrospective	III	Single-staged PSF	3/38 (41)	16.9 + 5.6	N/A
9	Grabala, 2020 [17]	Retrospective	III	Less-invasive TID followed staged pedicle screw instrumentation	3/19 (22)	14.8 ± 2.0	31
10	Gatam, 2020 [18]	Case series	IV	PSF	1/7 (8)	16.4 ± 1.8	12
11	Chan, 2021 [19]	Retrospective	III	PSF using a dual attending surgeon strategy	12/93 (105)	15.7 ± 5.0	N/A
12	Mihara Y, 2021 [20]	Retrospective	III	Single stage PSF with pedicle screw construct without any osteotomies	16/112 (128)	15.5 ± 4.5	N/A
13	Badin D, 2023 [8]	Retrospective	III	TID	1/17 (18)	13 ± 1.6	61.32
14	Stone LE, 2024 [21]	Prospective, multicenter	II	Combined anterior release with posterior instrumentation	3/13 (16)	14.4 ± 2.0	24
15	Grabala P, 2024 [22]	Retrospective	III	Halo gravity traction with PSF	2/18 (20)	16.5 ± 3.5	42
				Minimally invasive TID technique followed by staged surgery and PSF	10/32 (42)	16.4 ± 4.8	42
16	Chandirasegaran, 2025 [23]	Retrospective	III	Single-staged PSF	0/37 (37)	15.7 ± 3.4	N/A
*Ponte Ostetotomy/Smith Peterson Osteotomy*							
17	Zhang, 2019 [24]	Retrospective	III	Multiple-level asymmetrical Ponte osteotomies	5/21 (26)	26.7 ± 8.4	30.24 ± 10.6
18	Di, 2020 [25]	Retrospective	III	A two-staged posterior correction, using a temporary MCGR	5/12 (17)	14.5 ± 1.4	34.8
19	Koller H, 2021 [26]	Retrospective	III	Periapical release using advanced ponte osteotomies, segmental insertion of pedicle screws and a single MCGR	N/A (7)	15	19
20	Stone LE, 2024 [21]	Prospective, multicenter	II	Posterior with posterior column osteotomies	9/29 (38)	14.4 ± 2.0	24
*Vertebral Column Resection (VCR)*							
21	Zhou, 2011 [27]	Retrospective	III	Anterior and posterior VCR	8/8 (16)	16	32.4
22	Modi, 2011 [28]	Prospective	II	Posterior multilevel vertebral osteotomy	4/3 (7)	23.9	40
23	Ren, 2014 [13]	Retrospective	III	Anterior vertebral column resection followed by posterior VCR and instrumentation	9/17 (26)	15.1 ± 3.7	28.4 ± 4.6
24	Li, 2019 [29]	Retrospective	III	Anterior and posterior VCR	N/A (6)	15.5 ± 1.9	45.5
25	Zhang, 2019 [24]	Retrospective	III	Single-level posterior VCR	2/10 (12)	27.9 ± 7.5	30.24 ± 10.6
26	Song Z, 2022 [30]	Retrospective	III	Posterior VCR	N/A (87)	18.7	42
27	Stone LE, 2024 [21]	Prospective, multicenter	II	Posterior VCR	3/4 (7)	14.4 ± 2.0	24

M: Male; F: Female; N/A: not available; PSF = Posterior spinal fusion; TID = Temporary internal distraction; APSF = Anteroposterior spinal fusion; MCGR = Magnetically Controlled Growing Rod; VCR = Vertebral Column Resection.

### Outcome measurement and result

The smallest postoperative Cobb angle was recorded in the VCR group, 106.7 ± 9.7°, with a mean follow-up duration of 34.6 ± 7.9 months. Followed by the Ponte-Smith group (107.4 ± 10.5°) and the non-osteotomy group (107.0 ± 9.1°).

The correction rate was highest in the VCR group (62.1%), followed by the non-osteotomy group (61.5%) and the Ponte-Smith group (60.3%).

The lowest EBL was observed in the Ponte-Smith group, with a mean of 1164.1 ± 467.2 mL. Table 2 summarizes the EBL and the preoperative and postoperative Cobb angles from the included studies.

**Table 2:** Outcome of the study: Estimated blood loss (EBL), Preoperative, Postoperative, and Follow-up Cobb angle.

No	Author, years	EBL (mL)	Preoperative Cobb angle (°)	Post-operative Cobb angle (°)	Follow-up Cobb angle (°)
*Non-osteotomy*					
1	Shen, 2006 [10]	845.8 ± 293.5	99.08 ± 14.44	41.92 ± 14.05	46.4 ± 12.6
		666.7 ± 188.7	98.50 ± 9.41	39.92 ± 10.76	46.1 ± 11.2
2	Hamzaoglu, 2008 [11]	3000	122	60	56
3	Li, 2009 [9]	1648	97.9	50.5	53.7
4	Zheng, 2013 [12]	624 ± 216	102.2 ± 8.9	29.7 ± 5.9	32.1 ± 5.6
5	Ren, 2014 [13]	1,319.1 ± 608.2	104.8	25.1 ± 13.1	26.5 ± 12.0
6	Mihara, 2020 [14]	1593.5	104.4	46.4	N/A
7	Mirzashahi, 2020 [15]	660	97.58	34.88	N/A
8	Chan, 2020 [16]	2583,1	110.8 ± 12.1	54.4 ± 12.8	N/A
9	Grabala, 2020 [17]	1396	120	58	59
10	Gatam, 2020 [18]	N/A	103	34	N/A
11	Chan, 2021 [19]	1612.2 ± 873.5	104.5 ± 12.3	42.5 ± 13.5	N/A
12	Mihara Y, 2021 [20]	N/A	102.8 ± 12.3	44.4 ± 13.5	N/A
13	Badin D, 2023 [8]	N/A	99.3 ± 11.2	17.9 ± 8.9	17.4 ± 7
14	Stone LE, 2024 [21]	1700	112	36	N/A
15	Grabala P, 2024 [22]	588	124 ± 10.8	45 ± 13.8	44.9 ± 12.2
		740	122 ± 9.8	37.4 ± 11.4	40.9 ± 11.8
16	Chandirasegaran, 2025 [23]	1064.6 ± 473.3	101.8 ± 11.3	44.9 ± 12.1	N/A
Mean		1366.1 ± 724.1	107.0 ± 9.1	42.3 ± 13.4	42.3 ± 13.4
*Ponte Ostetotomy/Smith Peterson Osteotomy*					
17	Zhang, 2019 [24]	842.3 ± 426.3	98.5 ± 16.5	44.1 ± 17.7	44.9 ± 18.2
18	Di, 2020 [25]	950	98.2° ± 6.9	38.3° ± 3.0	40.1° ± 4.1
19	Koller H, 2021 [26]	N/A	118°	38.5°	39.6
20	Stone LE, 2024 [21]	1700	115°	49°	N/A
Mean		1164.1 ± 467.2	107.4 ± 10.5	42.5 ± 5.1	41.5 ± 2.9
*Vertebral Column Resection (VCR)*					
21	Zhou, 2011 [27]	1,916	99.3 ± 5.5	32.9 ± 12.3	34.3 ± 12.1
22	Modi, 2011 [28]	3015 ± 1213	106.1 ± 30.2	46.6 ° ± 12.7	46.4 ± 11.4
23	Ren, 2014 [13]	1712.5 ± 807	101.3	33.1 ± 10.8	32.9 ± 12.7
24	Li, 2019 [29]	1333.3 ± 574.2	108.91 ± 16.6	56.49 ± 18.8	56.9 ± 18.4
25	Zhang, 2019 [24]	1105	96.6	30.9	32.4
26	Song Z, 2022 [30]	N/A	108.7 ± 24.5	36.2° ± 12.4°	N/A
27	Stone LE, 2024 [21]	1650	126	48°	N/A
Mean		1788.6 ± 666.3	106.7 ± 9.7	40.6 ± 9.8	40.6 ± 10.8

EBL: Estimated blood loss; N/A: not available.

The Functional Outcome Score (SRS-22R) was highest in the non-osteotomy group, with a mean score of 4.3 reported in four studies, followed by the VCR group with a mean score of 4.1 from two studies, and the Ponte-Smith group with a mean score of 3.75 from two studies.

Several complications were identified in the study and were categorized according to the affected organ systems. The highest complication rate was observed in the VCR group, followed by the non-osteotomy and Ponte-Smith groups (24%, 19.6%, and 4%, respectively). A detailed breakdown of complications is presented in Table 3.

**Table 3:** Intraoperative and postoperative complications following severe scoliosis correction surgery.

Complications	Non-osteotomy	Ponte osteotomy/Smith Peterson osteotomy	Vertebral Column Resection (VCR)
	16.9%	4%	24%
*Massive blood loss*	3 [19, 20, 24]	–	–
*Wound*: Superficial infection, deep infection, wound abscess, wound dehiscence	18 [10, 11, 14, 16, 19, 20, 24, 32, 33]	3 [34]	6 [31, 34]
*Neurological*: Intraoperative neuromonitoring changes, anesthesia of the extremity, seizure, loss of SSEP	25 [14, 19, 20, 23, 24, 33]	1 [34]	1 [34]
*Respiratory*: Pneumonia, lung collapse, atelectasis, dyspnea, hematothorax, hematopneumothorax, pleural effusion, need for ventilator support	15 [13–16, 19, 21, 24, 33]	1 [35]	12 [13, 30, 31, 36, 37]
*Gastrointestinal and urinary tract*: Transient urinary retention, ileus	4 [15, 16, 21]	–	–
*Implant/fixation related*: Pin infection, screw loosening, pedicle screw were abandoned, pedicle screw malposition, titanium mesh cage malposition, screw breakage, screw prominence	11 [21, 32, 33]	–	2 [13, 30, 36, 37]
*Cardiovascular/circulatory*: SMAS, irreformable hypotension	7 [14, 19, 23, 33]	–	6 [31]
*Others*: Thermal injury, neck or back pain during traction, SIADH, soft tissue pain, rib hump deteriorated, optic deficit	19 [10, 15, 33]	1 [34]	3 [31, 34]

SSEP: somatosensory evoked potential, SMAS: superior mesenteric artery syndrome, SIADH: syndrome of inappropriate anti-diuretic hormone.

All patients from 10 studies reported being highly satisfied with the results, including seven studies in the non-osteotomy group, two in the Ponte-Smith group, and one in the VCR group. Table 4 provides a comprehensive summary of all results.

**Table 4:** Summarizes of the results.

	Mean EBL (mL)	Mean preoperative Cobb angle (°)	Mean Post-operative Cobb angle (°)	Correction rate (%)	Complications rate (%)
Non-osteotomy	1366.1 ± 724.1	107.0 ± 9.1	42.3 ± 13.4	61.5	16.9
Ponte Ostetotomy/Smith Peterson osteotomy	1164.1 ± 467.2	107.4 ± 10.5	42.5 ± 5.1	60.3	4
Vertebral Column Resection (VCR)	1788.6 ± 666.3	106.7 ± 9.7	40.6 ± 9.8	62.1	24

## Discussion

Scoliosis affects approximately 0.5–5% of the adolescent population, with idiopathic cases accounting for up to 80% [1, 2]. Severe scoliosis is typically defined by a primary curve greater than 90° and a flexibility of less than 25% on bending radiographs [3]. This systematic review of severe idiopathic scoliosis found that the VCR technique is the most effective procedure. It has the lowest postoperative Cobb angle, the highest correction rate, and the highest complication rate. The Ponte-Smith group has the lowest EBL and complication rate, and the non-osteotomy group has the highest functional outcome score.

This systematic review has several limitations. First, the majority of included studies were retrospective in nature and subject to inherent selection and reporting biases. Only two randomized controlled trials were identified, one of which had a high risk of bias. Second, there was considerable heterogeneity in surgical techniques, instrumentation, and perioperative protocols across the studies, which may confound comparisons. Third, not all studies consistently reported key outcome measures, such as estimated blood loss, complication types, and functional outcomes, which limited the ability to perform a comprehensive quantitative synthesis or meta-analysis.

The highest correction rate was documented by Badin et al. in 2023 [8], who reported that patients with severe scoliosis (mean preoperative major curve Cobb angle of 99.3 ± 11.2°) treated with TID achieved a coronal plane correction rate of 82%. The majority of studies [11, 14, 16, 17, 19–21, 23, 24, 28] on the surgical management of severe adolescent idiopathic scoliosis (AIS) report modest correction rates, typically below 60%, regardless of the surgical technique employed. However, the primary goal in these cases was not to achieve maximal correction, but rather to attain an acceptable overall spinal balance without significant complication. This is consistent with a previous meta-analysis involving 640 patients who underwent one-stage posterior spinal fusion for severe AIS, which reported a coronal correction rate of 58.6% for the major curve [34]. Posterior VCR is the most effective procedure for inducing effective correction compared to non-osteotomy and Ponte-Smith osteotomy procedures. The safe correction rate of the spine is based on the maximum displacement of the spinal cord that can be tolerated without causing damage [30]. A systematic review of seven studies, which included both pediatric and adult scoliosis patients undergoing the VCR surgical technique, reported a mean correction rate of 61.2% [30]. This is consistent with the current systematic review, which found a correction rate of 62.1% for the VCR procedure. Ponte osteotomy, which relies on posterior column shortening and anterior column lengthening, provides more modest correction (5–10° per level) and is most appropriate for flexible deformities in patients with preserved anterior disc mobility. Ponte osteotomy is a versatile surgical technique that can be safely and efficiently performed along the apex of a rigid spinal curve to improve flexibility and facilitate correction of gradual kyphotic or scoliotic deformities [24]. Based on an unpublished formal review conducted at our center involving 21 patients who underwent robotic-assisted correction for severe scoliosis, the mean correction rate was 59.2 ± 12.0%. A study by Pizones et al. [38] found that a Ponte osteotomy has a correction rate of 70% in 73 patients with AIS, specifically Lenke types 1–4. In contrast, Pedicle Subtraction Osteotomy (PSO) achieves significant sagittal plane correction (typically 30–40° per level) by resecting a wedge of the vertebral body through a posterior-only approach. While effective for fixed sagittal imbalance, such as in ankylosing spondylitis or flat-back syndrome, PSO is less suited for severe coronal deformity.

In the surgical management of severe scoliosis, particularly in cases involving rigid and multiplanar deformities, the choice of osteotomy technique plays a crucial role in achieving optimal correction and spinal balance. VCR offers the most powerful corrective potential by enabling simultaneous correction in both the coronal and sagittal planes. This technique involves complete excision of a vertebral segment, including its posterior elements and vertebral body, allowing for maximal mobilization and realignment of the spinal column [31, 39, 40]. VCR is particularly indicated in severe, rigid scoliosis – often exceeding 100° – such as in congenital deformities with structural vertebral anomalies or in revision cases where previous interventions have failed [40].

Numerous clinical studies have demonstrated the favorable safety profile of the Ponte osteotomy [41]. This technique is generally considered a less aggressive approach and is associated with reduced blood loss and lower overall surgical risk [42]. A retrospective study evaluating multiple asymmetrical Ponte osteotomies in severe and rigid idiopathic scoliosis found the technique to be safe and effective, with reduced operative time, blood loss, and complication rates [24]. A systematic review of nine studies reported an EBL of approximately 142.5 mL. This relatively higher EBL may be linked to the longer operative time typically required for the procedure, which can adversely affect clinical outcomes [43].

Untreated idiopathic scoliosis can lead to a gradual decline in quality of life, often resulting in chronic pain, reduced physical function, and psychological challenges, including depression, low self-esteem, and diminished self-confidence [44]. Two studies [45, 46] have demonstrated a strong inverse correlation between the degree of deformity correction and improvements in SRS questionnaire scores, indicating enhanced quality of life. However, patients often describe the surgical experience as challenging, likely due to factors such as postoperative pain, limited mobility, reduced trunk flexibility, and visible scarring. A previous study by Stone et al. [32] compared non-osteotomy and osteotomy approaches in patients with severe idiopathic scoliosis and found no significant difference in outcomes between the two techniques. However, both groups showed postoperative improvement across all domains. This study found that the non-osteotomy group had the highest SRS-22 scores, though the values were similar across all three techniques.

In a previous meta-analysis [43] involving 640 patients who underwent one-stage posterior spinal fusion for severe AIS, the overall complication rate was 5.4% and the major complication rate of 4%. The most common issues in the VCR procedure were respiratory complications, significant blood loss, neurological deficits, implant-related failures, and wound infections [31, 39, 40]. Management of perioperative complications requires timely and appropriate action. Wound complications, such as infections, abscesses, or wound dehiscence, are typically treated with antibiotics, proper wound care, and in some cases, surgical intervention [9, 14, 20]. Neurological complications, including changes in mental status or seizures, are managed through intraoperative adjustments, corticosteroids, or anticonvulsant medications. Respiratory problems, such as pneumonia, atelectasis, or pleural effusion, are treated with respiratory support, antibiotics, drainage when necessary, and mechanical ventilation in more severe cases [13, 15]. A key concern with combined anterior or posterior-only spinal fusion is the risk of pulmonary complications [47]. Anterior release has been specifically associated with greater impairment of pulmonary function compared to the posterior-only approach, resulting in a marked decrease in forced expiratory volume (FEV) and FEV1 values that can persist for up to five years after surgery [33]. Additionally, preoperative traction is associated with a higher risk of perioperative complications, including pin loosening, pin tract infections, and cranial nerve palsies [35].

One study reported that the VCR technique carries a higher risk of pleural effusion, especially in pediatric patients [48]. In contrast, adult spinal deformity patients who underwent posterior VCR were observed to have a higher incidence of intraspinal anomalies. However, this association may be attributed to the presence of neurological risk factors, including angular and abrupt spinal curves, coexisting intraspinal anomalies leading to increased spinal cord tension, and preoperative signs and symptoms of neurological dysfunction [36]. To minimize this risk, one study suggests that preoperative traction and intraoperative adjustment of spinal cord tension contribute to improved neurological safety [30].

Our unpublished in-house research, involving 21 patients who underwent robotic-assisted severe scoliosis correction surgery, found a minor complication rate of 14.3%, including screw breakage, screw pull-out, and loss of somatosensory evoked potential intraoperatively. But all was managed conservatively without any additional surgery. Notably, the robotic group experienced fewer severe and no major complications compared to the free-hand technique described in this study. We attribute this improvement to the haptic feedback provided by the robotic system, combined with real-time trajectory guidance via the navigation monitor, both of which are critical in ensuring accurate pedicle screw placement and thereby minimizing the risk of serious complications [37]. Given its extensive correction capacity, VCR remains the gold standard for the most severe and rigid scoliotic curves, albeit with higher technical demands and complication risks. Its judicious use in selected patients can result in meaningful deformity correction, improved spinal alignment, and an enhanced quality of life [49].

## Conclusions

Severe idiopathic scoliosis requires a tailored surgical approach that balances the magnitude of correction with procedural safety and efficacy. In the current systematic review, VCR achieves the most excellent deformity correction but carries the highest risk of complications, while Ponte osteotomy offers a safer alternative with moderate correction.

## Data Availability

No datasets were generated or analyzed during the current study.
